# On the Evaluation of the Suitability of the Materials Used to 3D Print Holographic Acoustic Lenses to Correct Transcranial Focused Ultrasound Aberrations

**DOI:** 10.3390/polym11091521

**Published:** 2019-09-19

**Authors:** Marcelino Ferri, José María Bravo, Javier Redondo, Sergio Jiménez-Gambín, Noé Jiménez, Francisco Camarena, Juan Vicente Sánchez-Pérez

**Affiliations:** 1 Centro de Tecnologías Físicas, Universitat Politècnica de València, Camino de Vera S/N, 46020 Valencia, Spain; jobrapla@fis.upv.es (J.M.B.); jusanc@fis.upv.es (J.V.S.-P.); 2 Instituto para la Gestión Integrada de las zonas Costeras, Universitat Politècnica de València, Carretera Nazaret-Oliva S/N, 46730 Valencia, Spain; fredondo@fis.upv.es; 3 Instituto de Instrumentación para Imagen Molecular, Centro Mixto CSIC-Universitat Politècnica de València, Camino de Vera S/N, 46022 València, Spain; serjigam@fis.upv.es (S.J.-G.); nojigon@upv.es (N.J.); fracafe@fis.upv.es (F.C.)

**Keywords:** 3D printed lenses, focused ultrasound, transcranial ultrasound, single-element transducer, transcranial therapy

## Abstract

The correction of transcranial focused ultrasound aberrations is a relevant topic for enhancing various non-invasive medical treatments. Presently, the most widely accepted method to improve focusing is the emission through multi-element phased arrays; however, a new disruptive technology, based on 3D printed holographic acoustic lenses, has recently been proposed, overcoming the spatial limitations of phased arrays due to the submillimetric precision of the latest generation of 3D printers. This work aims to optimize this recent solution. Particularly, the preferred acoustic properties of the polymers used for printing the lenses are systematically analyzed, paying special attention to the effect of p-wave speed and its relationship to the achievable voxel size of 3D printers. Results from simulations and experiments clearly show that, given a particular voxel size, there are optimal ranges for lens thickness and p-wave speed, fairly independent of the emitted frequency, the transducer aperture, or the transducer-target distance.

## 1. Introduction 

Holography is a technique to reconstruct wave fields from the previous recording of their complete amplitude and phase information. Optical holograms have been widely developed in many scientific, technological, and even artistic applications; however, acoustic holograms have been notably less applied to interest cases. Most acoustic holographic techniques are based on phased arrays with a large number of transducers [1,2], requiring sophisticated electronic circuits highly sensitive to calibration inaccuracies. However, new advances in metamaterials and the design capabilities of the state of art 3D printers allow the development of passive structures capable of shaping complicated acoustic fields [3,4,5,6,7,8,9].

An ambit in which precise holographic reconstruction of acoustic fields has been gaining importance in recent years is in focused ultrasound for medical applications (FUS), mainly in non-invasive treatments where the ultrasound propagates through tissues with very different acoustic impedances, as is the case of transcranial propagation [8,9,10,11,12,13,14,15,16]. The first successful transcranial ablation of animal brain tissue was achieved by Fry and Goss in 1980 [16]. Since then, many clinical procedures have been improved by applying transcranial focused ultrasound. Treatment of brain tumors [17,18], Alzheimer’s disease [19], or Parkinson’s disease [20] can be enhanced by this technique. The administration of FUS allows a local transient opening of the blood-brain barrier (BBB) [13,14,15,16,17,18,19,20,21], improving the delivery of pharmacological substances such as anticancer therapeutic drugs [22], neurotrophic factors [23], adeno-associated viruses [24,25], and neural stem cells [12]. 

The success of these medical treatments is linked to an adequate reduction of skull-induced aberrations to achieve a precise ultrasound focusing. Early approaches included ultrasonic therapy after craniotomy [26] or less aggressive techniques such as the application of FUS without correction of aberrations, but radiating from regular and flat areas of the skull [27]. More recent approaches mainly rely on actively shaping the wavefront to correct bone-induced aberrations through the use of holographic lenses or multi-element transducer arrays whose phase can be adjusted individually. The required phase registration is performed by inverse propagation, measured or simulated, from target to transducer. Initially, phase patterns were assessed by physically placing a reference transducer inside the brain at target location [28,29]. Presently, the technique is absolutely non-invasive as acceptable phase patterns can be obtained by numerical simulation [14,30,31]. 

Research which aims to improve the technique can be based both on (i) a better recording of the phase associated with the knowledge of the physical variables of each element of volume scanned by medical imaging and with the numerical methods applied and on (ii) a better reconstruction of the recorded field. In this work we address the second issue. In fact, the aberration correction by multi-element phased arrays is subjected to technological limitations associated with the size and number of singular elements that can be implemented in the phased arrays. These limitations can be overcome by shaping the wavefront using high transparency 3D printed refractive ultrasound lenses [6,8]. In fact, the work published by Maimbourg et al. [6] demonstrates that the spatial resolution, associated with the voxel size achieved by the latest generation of 3D printers, dramatically improves the space resolution of phased arrays, leading to better focusing. Therefore, in addition to the actual race towards creating arrays with an ever-increasing number of elements, the holographic acoustic lens approach emerges as a promising technology that allows submillimetric phase correction with limited cost.

In this work, assuming the relevance that 3D holographic lenses can take for transcranial FUS treatments in the near future, we aim to systematically evaluate the physical properties that the polymers used in their elaboration must have in order to reconstruct the desired acoustic field in an optimal way. There are trivial aspects, as that the lens must be energetically efficient, allowing a great transmission of ultrasound. However, other aspects, such as those related to the shape of the lens and the sound speed in the polymer, which are intimately linked to each other, require a more detailed quantitative study. In fact, in previous works, polymers with lower [6] or higher [8,9] than water wave speeds have been indistinctly used to build the lenses without explicitly expressing a suitability criterion. In both slow and fast materials, the p-wave speeds condition the thickness and shape of the lenses. There are important aspects of lens quality associated with shape, such as (i) the validity of the theoretical approach that assumes the hypothesis of ideally thin lenses, or (ii) the effect of voxel size on the printed lens, since the relative discretization of the lens is more abrupt in thin lenses. In order to give a quantitative response, or at least a guide to solve these questions, a series of numerical simulations and a validation experiment are proposed, the results of which are discussed and justified below.

## 2. Materials and Methods

### 2.1. Physical Parameters Obtained from Computerized Tomographies

Numerical simulations were conducted using the data acquired by computerized tomography (CT) of a human head (freely available at the repository cancerimagingarchive.net for scientific purposes) with an interslice spacing of 0.63 mm and an in-plane spatial resolution of 0.49 mm. A 3D linear interpolation of the radiodensity known at each node of this parallelepiped mesh was performed to obtain a denser cubic simulation grid with a spatial step, *h* = 0.244 mm. Different excitation frequencies are applied in simulations, the highest being 760 kHz, so a ratio of λ/*h* > 8 at water is ensured, which introduces acceptable numerical phase error in the simulations [32]. 

The elastodynamic damped Equations (1) and (2) for isotropic solids are applied at the whole computational domain, since the dynamics of the wave propagation in fluids can be defined as a particular case of solid with null shear modulus *G*. A set of four independent parameters must be known at each computational node, these being (i) the density in equilibrium, *ρ*, (ii) the p-wave speed, *c*, (iii) the attenuation coefficient, α, and (iv) the Poisson′s ratio, *ν*. The two first parameters are obtained by means of the linear interpolations [6]:

(1) $$c(x,y,z)=c_{water}+\left(c_{bone}-c_{water}\right)\frac{HU(x,y,z)-HU_{\min}}{HU_{\max}-HU_{\min}}$$

(2) $$\rho (x,y,z)=\rho_{water}+\left(\rho_{bone}-\rho_{water}\right)\frac{HU(x,y,z)-HU_{\min}}{HU_{\max}-HU_{\min}}$$

where HU is the radiodensity in Hounsfield units, *ρ_water_* and *c_water_* are respectively the density and p-wave speed of water at 21 °C, and *ρ_bone_* and *c_bone_* are respectively the density and p-wave speed of cortical bone at that temperature. Radiodensity values out of the interval 0 to 2400 HU were set respectively to the saturation values expected for water and cortical bone [33]. In accordance with the bibliography [6,34,35,36] the values considered for water and cortical bone are as follows: 

$c_{bone}=3100\;m/s$, $\rho_{bone}=1900\;kg/m^{3}$, $c_{water}=1485\;m/s$, $\rho_{water}=10^{3}\;kg/m^{3}$

Since the dependence of radiodensity on attenuation coefficient and Poisson′s ratio has not been investigated in depth, we have applied a simplified approach for these parameters, obtaining an average value at each domain. For the attenuation coefficients at 760 kHz we have taken α*_brain_* = 2 Np/m and α*_skull_* = 60 Np/m. [8,34,37,38], and Poisson′s ratio at the skull has been defined as uniform with a value ν*_skull_* = 0.316 to achieve a constant relation between p-wave and s-wave speeds, *c_p_/c_s_* = 27/14, as proposed by Hughes et al. [39].

### 2.2. Governing Equations and Numerical Model

A linear centered elastodynamic model in finite differences in time domain (FDTD) was developed by the authors in MATLAB (The MathWorks Inc., MA, USA). The governing Equations (3) and (4) for both fluid and isotropic solid media, are as follows [8]:

(3) $$\frac{\partial \tau_{ij}}{\partial t}=(M-2G)\delta_{ij}\left(\nabla \vec{u}\right)+G\left(\frac{\partial u_{i}}{\partial x_{j}}+\frac{\partial u_{j}}{\partial x_{i}}\right)$$

(4) $$\rho \frac{\partial u_{i}}{\partial t}+\sigma \cdot u_{i}=\sum_{j}\frac{\partial \tau_{ij}}{\partial x_{j}}$$

where *u* is the particle velocity, *τ_ij_* are the components of the stress tensor, δ*_ij_* is the Kronecker delta, *ρ* is the density in equilibrium, *M* and *G* are respectively the p-wave and the shear moduli, and *σ* is an artificial absorption parameter generating an exponential space dependent attenuation in isotropic media. The value of *σ* is implemented to obtain the proper attenuation coefficient for both solid and fluid media, attending to the relation proposed by Ferri et al [8].

(5) $$\sigma =\rho \sqrt{{\left(\omega +\frac{2c^{2}\alpha^{2}}{\omega }\right)}^{2}-\omega^{2}}$$

where ω is the angular frequency, *c* is the p-wave speed, and α, in Nepers per meter, is the absorption at the media. Shear modulus, *G*, is obtained from its known relation with Poisson’s rate and p-wave modulus at solid media and is forced to zero in the fluid domain. 

Equations (3) and (4) are valid for both solid and fluid media. At liquid media, the null value of the shear modulus *G* leads to a null value of the tangential stresses and to an equal value of the three axial stresses at each point, equivalent to the fluid pressure with the opposite sign. The parameter *M* respectively represents the p-wave modulus at the solid domain and the bulk modulus at fluid and it is obtained at any point as follows:

(6) $$M=\rho c^{2}$$

The excitation signal, for both time forward and time reversal simulations, consists of a harmonic pressure excitation enveloped by a half Hanning window during the first *n* cycles, with *n* = 8. Excitation pressure is implemented in complex form, in order to facilitate the phase computation at the holographic registration surface, as follows:

(7) $$\tau_{11}=\tau_{22}=\tau_{33}=-p_{o}\sin^{2}\left(\frac{\omega }{4n}\min\left(t,\frac{2n\pi }{\omega }\right)\right)\left(\cos\left(\omega t\right)+j\sin\left(\omega t\right)\right)$$

The time step Δ*t* is adjusted to a 0.075 Courant–Friedrichs–Lewy (CFL) condition at water that ensures stability at the fastest evaluated holographic lens that is faster than cortical bone and four times faster than water. The definition of a CFL linked to the water, instead of dependent on the fastest media as usual, attends to the fact that the numerical isotropy of the wave speed is CFL dependent [40]. Its oversized value intends to avoid favoring propagation in some lenses over others, as it is far from the optimum in all cases. 

### 2.3. Holographic Phase Pattern Registration 

**Figure 1:**
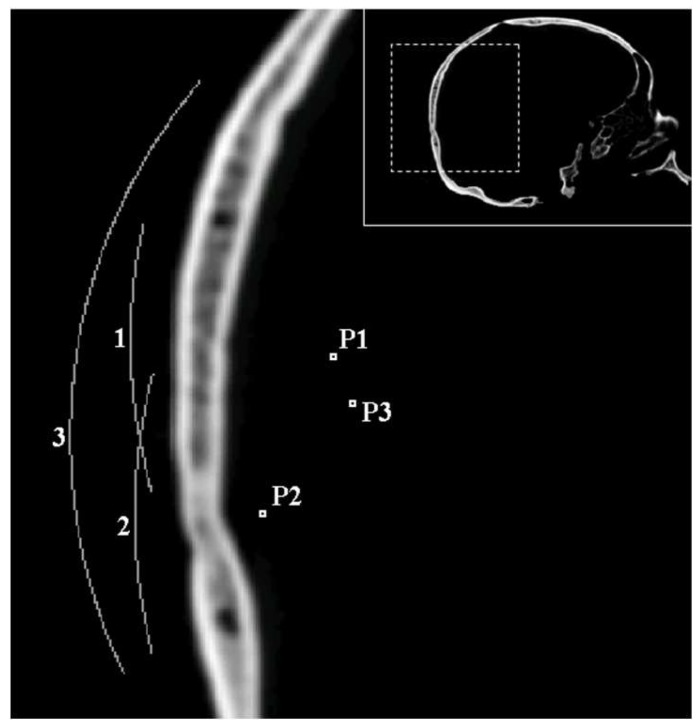
Schematic sagittal cross section of the skull, representing the positions of target points (**P1**, **P2**, **P3**) and registration surfaces (**1**, **2**, **3**) considered in numerical simulations.

The lens shape is derived from the phase pattern obtained by simulation at the holographic registration surface, concentric to the surface where the transducer must be placed, at a distance equivalent to the nominal lens thickness (Figure 1). Properly, the holographic registration surface is a Cartesian discretized spherical or plane surface where the phase is obtained from the value of the pressure in a complex form known at the Cartesian nodes of the computational grid. This pressure field is simulated by the time reversal propagation of a single frequency wave emitted at the target point. At the stationary state, achieved several periods after the wave reaches the registration surface, the numerically computed phase shift between any two points of the computational domain remains remarkably constant. Phases calculated at the holographic registration surface are not subjected to a process of unwrapping, as recommended in previous works [6,8], since this work is focused on evaluating holographic Fresnel lenses capable of a maximum phase shift of 2π. Instead, we just add an arbitrary value to the phase pattern to reduce the region of the lens affected by an abrupt 2π phase shift.

### 2.4. Numerical Design of Holographic Lens

The thickness at each point of the lens defined in spherical coordinates, *h*(θ,φ) is obtained by linear interpolation from the phase β(θ,φ) computed at the holographic registration surface, as follows:

(8) $$h(\theta ,\phi )=\frac{\beta (\theta ,\phi )}{2\pi }d\;\mathrm{or}\;h(\theta ,\phi )=\frac{2\pi -\beta (\theta ,\phi )}{2\pi }d$$

where the first expression is used for lenses made with materials with wave speed smaller than water and the second for fast lenses, and where *d* is the nominal lens thickness obtained for both fast and slow lenses, as a function of its relative speed (*c_rel_* = *c_p_lens_*/*c_water_*), as follows:

(9) $$d=\left|\frac{c_{rel}}{c_{rel}-1}\right|\lambda_{water}$$

where λ_water_ is the wavelength of the surrounding media (generally water) and the relative speed is obtained from the p-wave speed of the lens. The phases, β(θ,φ) required to obtain the thickness pattern of the lens, *h*(θ,φ), are computed after interpolating the phases, β(*x*,*y*,*z*), at the Cartesian holographic registration surface to the closest points of a mesh defined in spherical coordinates centered at the transducer focus. If 3D lenses are implemented in simulations, their shapes are linearly interpolated back to the Cartesian domain.

Linear interpolation proposed in Equation 8 is accurate if no inner reflections in both surfaces of the lens are considered, i.e., if the acoustic impedance of the lens is reasonably similar to the acoustic impedance of the surrounding media (water). If this hypothesis of total transmission is not acceptable, the thickness of the lens should be obtained applying the Fabry–Pérot approach, as proposed by Jimenez-Gambín et al [9]. This approach attends to an implicit expression that should be solved numerically for each value of β(θ,φ). The relevance of the systematic error assumed by applying the explicit linear interpolation is discussed in later sections.

### 2.5. Experimental Equipment and Materials

**Figure 2:**
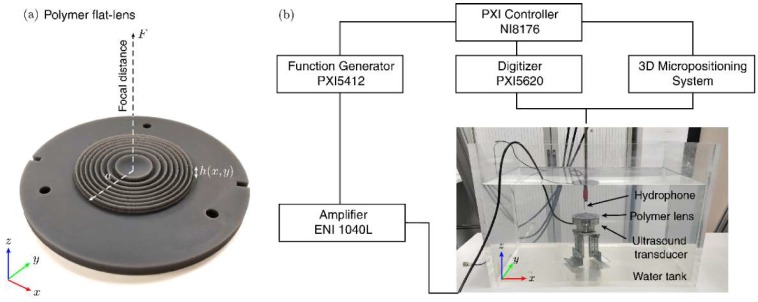
(**a**) Photograph of the flat-lens manufactured by a stereolithographic 3D-printing technique using a photopolymer. (**b**) Scheme of the setup and equipment used for the experiments in water tank.

The acoustical lens employed at the validation experiment was manufactured by stereolithographic 3D-printing techniques (Forms 2, Formlabs Inc., Somerville, MA, USA) using a photopolymer resin (Grey Standard Form 2, Formlabs Inc., Somerville, MA, USA) with a resolution of 100 μm (axial) and 140 μm (lateral), as shown in Figure 2a. The material was post-cured after the 3D printing process with a 1.25 mW/cm² of 405 nm LED light for 30 min at 60 °C. The acoustical properties of the material were obtained experimentally using a pulse-echo technique in a test cylinder with a height of 30 mm and a radius of 25 mm, resulting in a measured sound speed of *c_p_* = 2440.7 ± 2.2 m/s and a density of *ρ* = 1162.0 ± 1.4 kg/m^3^; as listed in Table 1 where the data of a set of 3D printable polymers are presented [41,42,43,44,45]. 

The rest of devices applied consist of a single-element piezoelectric transducer (PZT26 Ferroperm Piezoceramics, Kvistgaard, Denmark), a signal generator (PXI5412, National Instruments, Austin, TX, USA) amplified by a linear RF amplifier (1040L, ENI, Rochester, NY, USA), a needle hydrophone (HNR-500, Onda, Castellón, Spain) calibrated from 1 to 20 MHz, a digitizer (PXI5620, National Instruments, Austin, TX, USA), a thermistor probe (Tinytag Temperature Logger TK 4023, Dundee, UK), and a precision 3D micro-positioning system (OWIS GmbH, Staufen, Germany) All the signal generation and acquisition processes were based on a NI8176 National Instruments PXI-Technology controller, which also controlled the micro-positioning system.

### 2.6. Sources of Errors in the Printed Lenses

The main causes of error in the correction of the aberration, assuming a perfect application of the technique (i.e., disregarding any error associated with the position and orientation of the lens once printed), are described here. Even supposing an exact knowledge of the physical parameters at each point of the brain and skull and a with set of equations accurately describing the dynamics of the wave, there would be two inevitable sources of error affecting the computational modelling of the lens, as follows: Those relative to (i) the discretization of the domain and (ii) the theoretical simplifications assumed in the design of the lens shape starting from the registered hologram.

Regarding domain discretization, given the dependence between the accuracy of any numerical method and its computational cost, the only way to eventually achieve a null error would be with an infinite computational cost [46]. On the other hand, obtaining the shape of the lens from the registered hologram presents several difficulties. In the definition of the lens thickness as a function of the phase pattern recorded at the holographic surface -either by means of the Fabry–Pérot resonator approach [9] or by means of the proposed linear interpolation. The hypothesis of an idealized propagation of each ray in a radial direction (transducer-center) from the transducer to the holographic surface is assumed. This hypothesis, which is assumed to define the shape of the lens from the simulation, is incorrect. In fact, any real ray that crosses the limit between two different media changes its direction at the boundary (defined in this case as the periphery of the lens), but in time reverse simulations we assume that the direction is radial between the holographic surface and the transducer. This implies that an eventual change in the direction of the ray would be modeled as produced on the holographic surface and not on the periphery of the lens, as should be expected. In any case, the thinner the lens, the smaller the distance between the periphery of the lens and the holographic surface and, therefore, the more acceptable the hypothesis will be. The assumption of a linear relationship between the registered phase and lens thickness, rather than applying a more accurate model, is a second cause of error associated with this item.

In addition to the two aforementioned causes of error, exclusively associated with the numerical simulation that concludes obtaining the CAD model of the lens as an STL file, the process of generating a physical lens starting from the CAD model is affected by three new error sources, as follows: (i) Inaccuracy of shape, (ii) discretization of the shape, and (iii) imprecision in the physical parameters that affect wave propagation, such as density, Young′s modulus, Poisson′s ratio, or acoustic absorption associated with the presence of anisotropy, porosity, heterogeneity, etc., related with the printing process. The inaccuracy of shape is considered negligible in this study, since updated 3D printers can generate decimeter objects with inaccuracies of less than one tenth of a millimeter. As for the discretization of the shape of the lens, the dimensions of the elementary volume cell (voxel) depend on the 3D printing method (FDM, SLA, etc.) and the material used. Furthermore, given that 3D printing is a booming sector, the minimum voxel size is continuously being reduced. For the present study we accepted a cubic voxel with a side of 0.244 mm, since it is a reasonable value that matches with the grid element used in numerical calculations. Regarding the third cause of error, it should be noted that the anisotropy of the printing process itself (which is carried out in layers) can lead to large anisotropies in the value of the propagation speed, which is the most relevant parameter for the proper performance of these lenses. In this sense, it can be highlighted that if resins with fibers are used in FDM printing, we can find large anisotropies, such as compressibility modules of respective values *B*_x_ = 10 GPa and *B*_YZ_ = 1.12 GPa [41]. In general, SLA printing with subsequent curing using fiberless resins is the process that guarantees greater isotropy, although a certain degree of anisotropy or heterogeneity in the physical values of the printed lens can never be neglected [42,43,44,45].

Summarizing, five sources of error have been considered, as follows: Discretization of the numerical domain, validity of the theoretical approach, inaccuracy in the form of the printed physical lens, discretization of the printed lens, and imprecision in the relative p-wave speed of the printed lens. Between these, the inaccuracy in the form has been neglected and the size of the voxel associated with the printing process has been set to the size of the cell used for the numerical calculations. Thus, the three following sources of error were studied: Spatial discretization, imprecision on the polymer p-wave speed, and the validity of the theoretical approach.

The effect associated with the imprecision in the relative speed of the printed lenses, accepted their isotropy, can be approached as follows. The maximum phase shift produced by a lens with a nominal thickness, *d*, being *c*_rel_ the ratio between the p-wave speeds of the lens and the medium, is written as follows:

(10) $$\beta =\left|\frac{2\pi }{\lambda_{water}}-\frac{2\pi }{c_{rel}\lambda_{water}}\right|d$$

If the lens is made with a negligible error in its dimensions, the absolute uncertainty of the phase shift, ε(β), associated exclusively with the imprecision of the relative speed is obtained as follows:

(11) $$\varepsilon (\beta )=\frac{2\pi }{c_{rel}^{2}\lambda_{water}}d\cdot \varepsilon (c_{rel})$$

and knowing the value of the nominal thickness according to Equation (9), the phase error as a function of the relative error of the relative velocity, $\varepsilon_{r}(c_{rel})$, can be expressed as follows:

(12) $$\varepsilon (\beta )=\left|\frac{2\pi }{c_{rel}-1}\right|\varepsilon_{r}(c_{rel})$$

Regarding the effect on the phase shift associated to the spatial discretization of the lens, we can state that the maximum error of the phase shift, ε′(β), associated with the size of the voxel is written as follows:

(13) $$\varepsilon \prime (\beta )=\left|\frac{2\pi }{n_{d}}\right|$$

where *n_d_* represents the number of voxels associated with the nominal lens thickness. Thus, knowing the nominal thickness according to Equation (9) and since *n_λ_* is the number of voxels per wavelength in water, it is trivial to obtain the following:

(14) $$n_{d}=\left|\frac{c_{rel}}{c_{rel}-1}\right|n_{\lambda }$$

Combining the above Equations (13) and (14), we get the following:

(15) $$\varepsilon \prime (\beta )=\left|\frac{c_{rel}-1}{c_{rel}}\right|\frac{2\pi }{n_{\lambda }}$$

**Figure 3:**
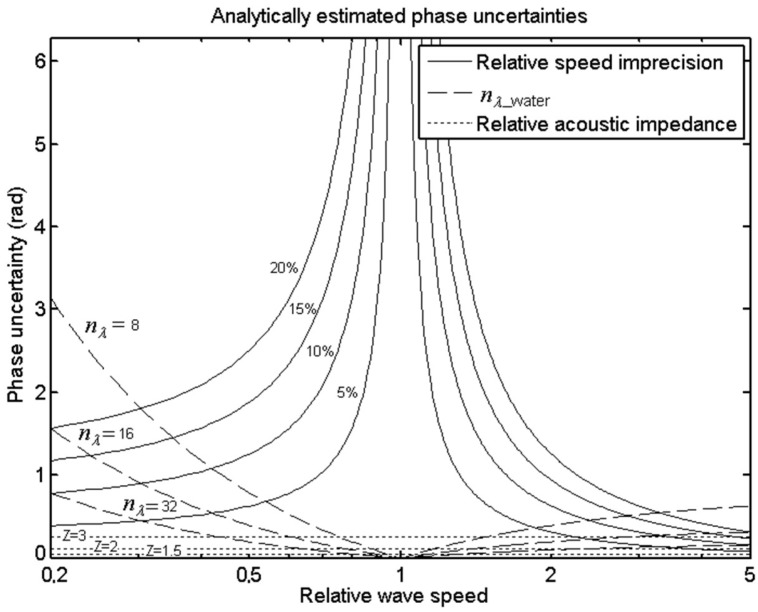
Analytical estimation of the uncertainties in the phase recreated by the 3D lens at the holographic surface associated with the discretization of the domain in voxels per water wavelength, *n_λ_water_*, (dashed), with the acoustic impedance of the lens when the definition of the thickness is approached by linear interpolation (dotted) and with possible differences between simulated and real values of the p-wave speed of the lens (continuous).

For a better understanding of the implications of Equations (12) and (15), Figure 3 is attached. It can be appreciated that if polymer p-wave speeds are close to that of water, the error associated with relative speed shoots towards infinity, whereas that associated with discretization tends to zero. It can be seen that the curves are asymmetrical, obtaining a more favorable trend for both errors in the high speeds zone.

The last error factor considered is the validity of the theoretical approach itself, which is affected by an accidental and a systematic error. The accidental error is associated with the difference between the actual ray path and the idealized path that tends to coincide in very thin lenses. This accidental error is difficult to quantify and is evaluated through a series of numerical simulations. In addition, a systematic error, associated with the estimation of the thickness of the lens by means of a simple linear interpolation and not by means of a more accurate model such as the Fabry–Pérot resonator [9], is introduced. The reasons for using the linear hypothesis in this work are simple, as follows: First, (i) both models converge when the impedances of water and lens are equal and the total transparency is reached (condition that has been applied in the set of numerical simulations evaluated), and second (ii), to apply the Fabry–Pérot resonator model, the acoustic impedances of the transducer, of the backing, and of the coupling agent must be known. This would enforce us to play with many input variables with a minor effect on the quality of the approach and therefore we dismissed this variability for the study. The maximum value of the systematic error in the phase associated with the assumption of the linear approach is represented in Figure 3. This error has been calculated as the worst in the whole phase interval (0, 2π) in the absence of coupling between the transducer and the lens by comparing the linear model with a Fabry–Pérot resonator.

Concluding, it can be stated that while the p-wave speed of each polymer affects lens shape and therefore the validity of the thin lens hypothesis, its acoustic impedance is not a direct cause of error in the phase generated at the registration surface. However, if this impedance is very different from that of water, transmission is decreased what is energetically inefficient. No study on optimal impedance has been proposed, therefore, as it is simple to conclude that the ideal lens would have an impedance equal to that of water.

## 3. Experiments and Simulations

In order to determine the suitability of the polymers attending to their p-wave speed, a set of numerical simulations and a validation experiments were performed and the details of which are described here. The obtained results are presented and discussed in later sections. 

### 3.1. Numerical Simulations 

The simulations were carried out with or without the skull interposed between the transducer and target, which were placed at different points (P1, P2, P3), always between the spherical transducer and its geometrical center (Figure 1). At each of these points and for each emitted frequency, we assumed the same protocol, as follows: First of all, the phase pattern was recorded (by means of time reversal simulation from target to transducer) on the holographic recording surface, both with and without the interposed skull. Then, in order to determine the characteristics of the ideal focus, the “gold standard emission”, which consists of emitting from the registration surface affected by the phase pattern recorded without skull, was simulated. Next, a series of six lenses for six different speeds slower than water and another series of six lenses for six speeds faster than water were numerically generated from both the phase patterns recorded with or without the skull. In the graphs and for the sake of brevity, we named concave or convex lenses the lenses produced respectively with fast or slow materials, since all the target points evaluated were located between the lens and its geometric center. The p-wave speeds evaluated ranged from one fourth to four times the wave speed of water.

**Figure 4:**
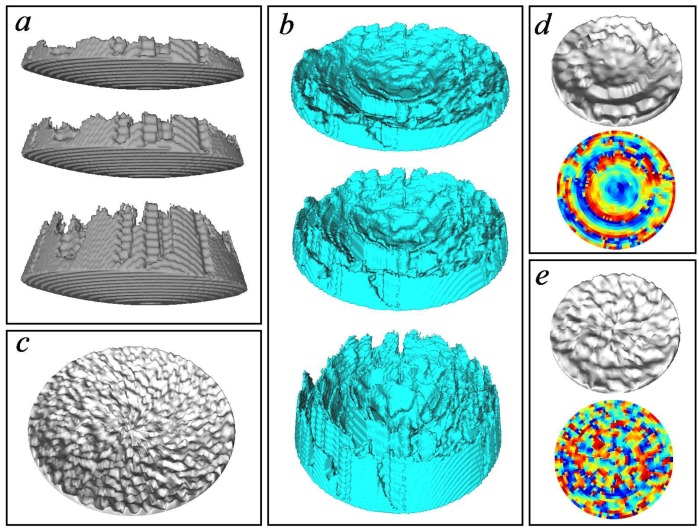
Representation of the Cartesian model of the lenses implemented in the simulations at points P1 (**a**) and P2 (**b**), with respective thicknesses of 10, 18, and 40 voxels. Images (**c**–**e**) represent the 10-voxel thick polar lenses associated respectively with points P3 (**c**) P2 (**d**) and P1 (**e**).

Once we calculated the lens at each configuration (i.e., at each point, at each frequency, with or without the skull) and for each p-wave speed of the lens polymer, the numerical medium was modified by adding each Cartesian model of the acoustic lens (Figure 4) placed in the correct position to generate the holographic pattern. Then, the time forward emission was simulated from the spherical transducer, emitting with uniform initial phase and amplitude, and placed concentric to the phase registration surface at a distance equivalent to the value of the nominal lens thickness. A backing with the same acoustic properties of the lens was added to avoid the numerical issues related with placing the emitting nodes at a boundary. 

The density of each simulated lens was established in accordance to its p-wave speed in order to set a unit normalized impedance for each polymer. Therefore, the simultaneous evaluation of velocity and impedance effects was avoided, but the properties of the simulated polymers could not correspond to any physical material.

For each of these simulations, the quality of the −3 and −6 dB focus beams was evaluated attending to seven quantitative indicators described below. The particular values used in the simulation series were the following: The p-wave speeds of the fast lenses were, respectively, 4, 2.5, 1.75, 1.5, 1.375, and 1.3 times the speed of water, whereas the p-wave speeds of the slow lenses were obtained dividing the speed of water by the same series of numbers. The spherical transducers had a radius of 59 mm and an aperture of 64 mm for configuration P3 and 30mm for P1 and P2. Figure 1 shows the target points whose respective distances to the holographic surface were 24.2 mm, 13.8 mm, and 30 mm. In short, there were 96 time forward simulations for the small aperture lens (two types of lens, two frequencies, with and without the skull, two positions, and six speeds) and 13 simulations for the lens of larger aperture.

### 3.2. Quantitative Focusing Indicators 

To assess the quality of the focal spot achieved by each kind of material, we calculated seven quantitative indicators, as described by Ferri et al. [8] for both −3 and −6 dB focal beams. For each focal beam, these indicators evaluated (i) its longitudinal and transverse deviations from the target point (*z*, *R*), (ii) its transverse and total gyradii (*k*_R_, *k*), (iii) orientation, ∆φ, (iv) the focal volume, *V*, and (v) energetic overlapping with the ideal focus, *I*_i_ (%). 

### 3.3. Experiments 

A validation of the simulation was performed by measuring the acoustical field of a 3D printed test lens in the ultrasonic regime. The experiments were done inside a 1 m × 0.75 m × 0.5 m degassed-distilled water tank at 21 °C, as shown in Figure 2b. The lens was excited using a custom-made ultrasonic source composed of single-element piezoelectric transducer mounted in a custom designed stainless-steel housing with a diameter 2*a* = 50 mm. The transducer was driven with a 50 cycles sinusoidal pulse burst at a frequency of *f* = 1.112 MHz. The pressure field was mapped by a 500 μm needle hydrophone. The hydrophone signals were digitized at a sampling rate of 64 MHz, averaged 50 times to increase the signal to noise ratio. A precision 3D micro-positioning system was used to move the hydrophone in three orthogonal directions with an accuracy of 10 μm. The scanned area covered from −3 to 3 mm in the *x* and *y* axis and from 10 to 35 mm in the *z* axis, using a step of 0.1 mm in all directions. Temperature measurements were performed throughout the whole process, by means of a thermistor probe placed at a distance of 5 cm of the focal spot, to ensure no temperature changes of 1 °C. To limit thermal effects not considered in the set of equations, we applied a sound pressure below 10 kPa at the focal beam, small enough to neglect nonlinear effects in the propagation [47], and a short burst of 50 cycles (*f* = 1.112 MHz) repeated each 1 ms, leading to a duty cycle of only 4.5%.

The properties of the material chosen to elaborate the axisymmetric lens were in the range of optimal values found in the simulations, which, in terms of nominal thickness, is defined between 10 and 25 voxels for any frequency as discussed below. The emission frequency applied in the experiment was higher than that established in the simulations (1112 and 760 KHz respectively). So, given that the nominal thickness is inversely proportional to the frequency, we found that the optimal material for this experiment, among those presented in Table 1, was Standard Grey®, since it has a nominal thickness of 14 voxels for this frequency and can be printed by stereolithography (SLA) that provides more homogeneity and isotropy than the fused deposition modeling (FDM) [42].

## 4. Results

This section presents the results of the set of simulations evaluated and the validation experiment carried out. Numerical parameterization of the focusing at each configuration simulated has been performed for both and −6 dB beams, however, given the similarity of the results obtained; only the indicators obtained at −6 dB will be presented. 

### 4.1. Emission in Water at 760 kHz

**Figure 5:**
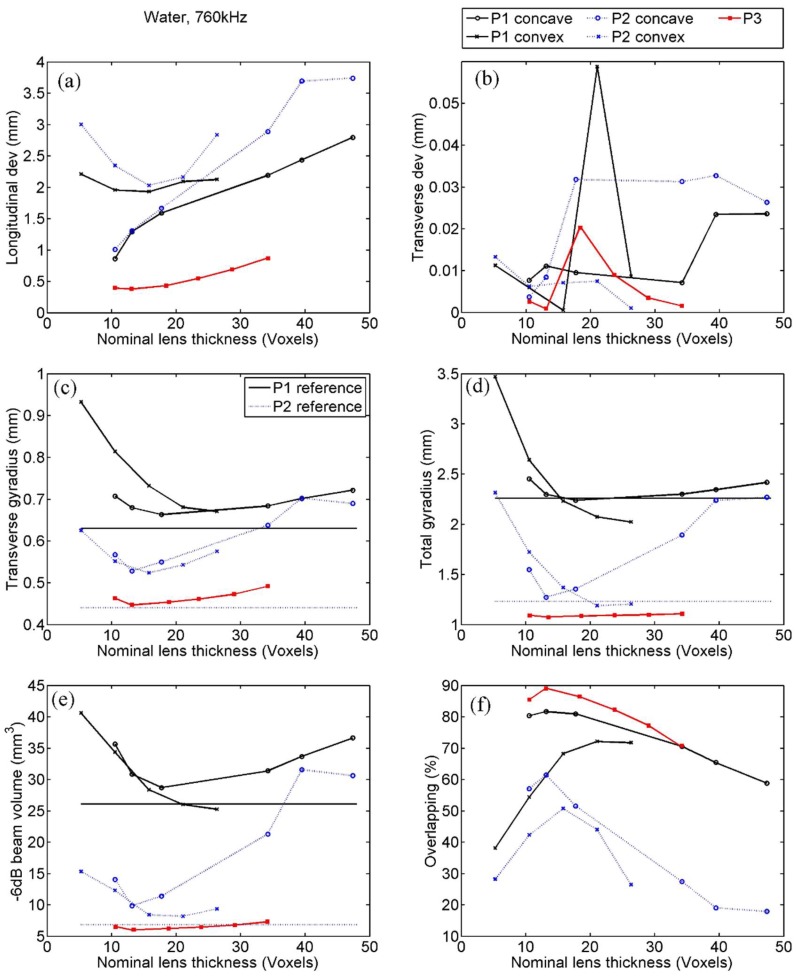
Focusing quality indicators calculated by simulation in water with 760 kHz, at points P1, P2, and P3, for concave (fast) and convex (slow) lenses. Indicators are as follows: (**a**) Longitudinal and (**b**) transverse deviation, (**c**) transverse and (**d**) total gyradius, (**e**) beam volume, and (**f**) energetic overlapping.

Positional deviation of the focus: The longitudinal focal point deviations of the −6 dB beam, for the simulations performed with each of the lens evaluated, are shown in Figure 5a. It can be seen that the trends found in the deviation values are different for concave and convex lenses, but somehow independent of the evaluated point. The obtained longitudinal deviations for the convex lenses seemed to present optimum values for nominal lens thickness between 10 and 20 voxels, whereas, for concave lenses at points P1 and P2, the results show that the thinner the better. The concave lens at point P3 exhibited an intermediate behaviour, with a flat trend between 10 and 15 voxels.

The transverse deviations found (Figure 5b) were negligible compared to the emitted wavelength (λ*_water_* ≈ 2 mm at 760 kHz) and to the size of the voxel for all the configurations evaluated, showing a maximum value of 60 μm, which is less than one third of the computational internode distance. No trend can be analyzed as the calculated transverse deviations simply showed a stochastic behaviour.

Gyradius: The proper beam shape, considered similar to the shape of the reference beam, was evaluated using the transverse, *k*_R_, and total, *k*, gyradii of the −6 dB focal beams (Figure 5c,d). The reference −6 dB beam obtained at P1 had the values *k*_R_ = 0.63 mm and *k* = 2.26 mm, and at P2 the values were *k*_R_ = 0.44 mm and *k* = 1.23 mm. Both the transverse and total gyradii at the −6 dB focal beams had certain similarities with those of the reference beam, at all the evaluated points and for concave and convex lenses. Particularly, and with the exception of a couple of cases of the total gyradius for convex lenses at both P1 and P2, the beams with interposed lenses were larger than the reference beam, as could be expected. Therefore, the smaller the gyradius, the more accurate the beam. Then, we found that concave lenses, as in positional deviation, showed its best results for nominal lens thickness between 10 and 20 voxels at both P1 and P2, whereas convex lenses seemed to present the best values with lightly thicker lenses for P1. At P2, however, the transverse gyradius presented a clear trend with an optimum between 10 and 20 voxels and the trend of the total gyradius behaved pretty like in P1. The concave lens at P3 showed relatively flat trends but an optimum range between 10 and 20 voxels could again be appreciated. 

Focal volume: The focal volume was compared with that of the reference beam and it was found that reference is always smaller, with values of 26.1 mm^3^ for P1 and 6.8 mm^3^ for P2. It is somehow obvious that the focal volume is highly linked with the gyradii values, so the trends found in Figure 5c–e present high similarities. As in gyradius, we found that, for concave lenses at the three points, the trend clearly signaled an optimum when the nominal lens thickness was between 10 and 20 voxels, and in convex lenses the optimum for both P1 and P2 was found at slightly larger values 

It is also remarkable that the most accurate values presented a great similitude with the reference beam, with differences as slight as 1% whereas the thickest or thinnest lenses present values with differences with the reference beam as large as 56%.

Orientation: No plot of orientation was presented, as their average values in water were smaller than 0.009 rad (0.5°). This indicator here only informs about numerical uncertainty, as in the case of transverse deviation. 

Energetic overlapping: Figure 5f shows that the largest overlaps for −6 dB beams were achieved at point P3 and P1 with concave lenses, with values as big as 89%. Concave lenses exhibit a slight better behaviour than convex ones for both points P1 and P2, and P1 shows better overlapping than P2 regardless of the kind of lens. The trends at the five curves show similarities with those observed in previous parameters such as focal volume and gyradii. The best overlaps were found with a lens thickness between 10 and 20 voxels for all the cases, with the only exception being convex lenses at P1, where the optimum was found between 15 and 25 voxels.

### 4.2. Transcranial Emission at 760 kHz

**Figure 6:**
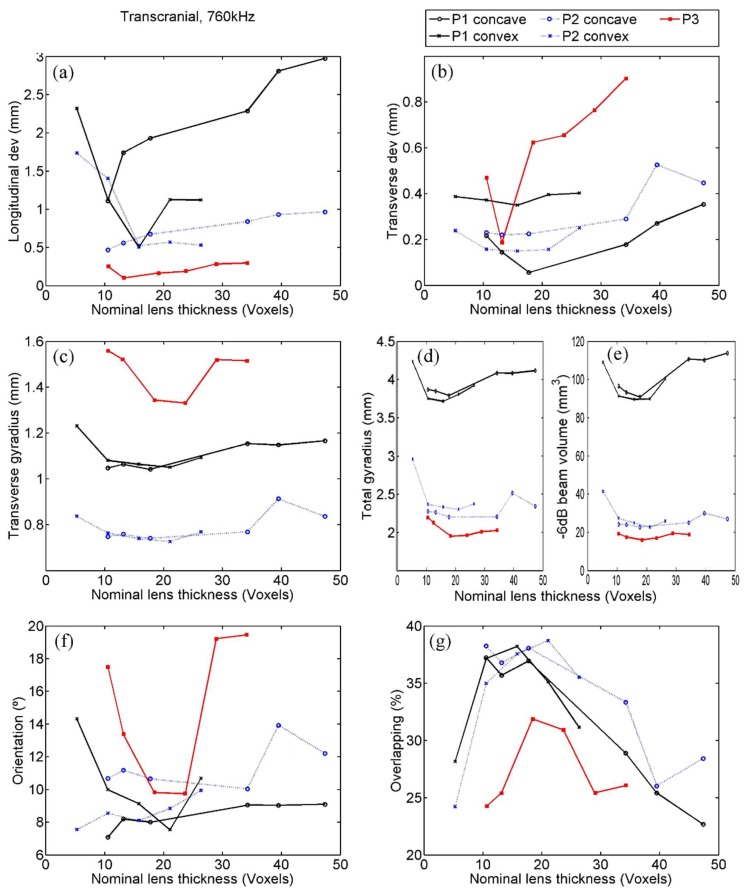
Focusing quality indicators calculated by transcranial simulation with 760 kHz at points P1, P2, and P3, for concave (fast) and convex (slow) lenses. Indicators are as follows: (**a**) Longitudinal and (**b**) transverse deviation, (**c**) transverse and (**d**) total gyradius, (**e**) beam volume, (**f**) orientation, and (**g**) energetic overlapping.

Positional deviation of the focus: In transcranial emission both longitudinal and transverse focal point deviations are relevant, and the values for the −6 dB beams are shown in Figure 6a,b. The transverse deviations obtained were relatively small compared to the emitted wavelength (λ*_water_* ≈ 2 mm at 760 kHz) and to the size of the voxel in the evaluated cases for each of the thicknesses, points, and types of lens. Their values, under 0.6mm in all the cases, were notably smaller than the values of the longitudinal deviation, which was less than 3 mm in all the simulations.

Despite the small values found for transverse deviations, the trends can be easily appreciated and were notably similar than those found in previous indicators, such as gyradius or focal volume in the underwater case. Again, the smallest deviations for concave and convex lenses at the points P1 and P2 were obtained in the interval between 10 and 20 voxels. Point P3 exhibited an aggressive trend with a minimum around 15 voxels. The smallest deviations at P1 were achieved with concave lenses and with convex ones at P2.

For longitudinal deviation, there was some similitude between the trends obtained for the underwater and the transcranial case. In transcranial case, trends were less explicit, but show again that the optimum nominal thickness for the convex lens was found between 10 and 20 voxels at P1 and bigger than 10 voxels at P2, whereas, for concave lenses at points P1 and P2, it was found that the thinner the better. At point P3 the optimal interval was again between 10 and 20 voxels. 

Gyradius: The transverse and total gyradii of the −6 dB focal beams are shown at Figure 6c,d. A great concordance between the data at both plots can be highlighted for points P1 and P2, since the trends of transverse gyradii are almost exactly proportional to those of the total gyradii for any type of lens. On the contrary, point P3 shows transverse gyradii values notably larger than expected. The values at the three points, for any type of gyradius at any simulation, were again slightly bigger than the reference values, as displayed in Figure 5c,d. The similitude between the results found with concave and convex lenses for both P1 and P2 is notable. However, it is difficult to find conclusive results about the preferable nominal thickness, as the curves are quite flat. At point P1 we could appreciate that the smallest gyradii with convex lenses were in the interval between 15 and 25 voxels, but in the rest of the cases we just can affirm that this interval was not worse than any other.

Finally, by comparing these data with those found at the underwater case, we can appreciate that the transcranial aberrated corrected beam was slightly larger, indicating that the dispersion of the energy in the skull was not fully compensated by the lens. This effect was particularly appreciable in the transverse gyradius at point P3.

Focal volume: In brief, we can summarize that the results found for the focal volume were in great accordance with that of both gyradii (Figure 6e). Thus, convex and concave lenses presented similar results between them and the trends found in transcranial and water cases were also very similar. What can be highlighted is that the aberrated corrected beams were notably larger than those found in the water case. This difference could seem excessive if compared with that found at gyradii but this apparent incoherence is associated with (i) the different units of gyradius and volume so the relative uncertainties in volume are three times bigger than those of gyradius and, additionally, (ii) the central part of the beam is reasonably similar between transcranial and water cases, but as we separate from the target point there is more diffuse energy in the transcranial case and an important fraction of this diffuse energy exceeds the arbitrary threshold of −6 dB. Finally, the interval between 10 and 25 voxels for the lens thickness was associated with the best results at P1 and this fact is not in contradiction with the flat trends found at P2 and P3. 

Orientation: This indicator, as has been defined, is highly sensitive to small asymmetries or irregularities associated to a non-exact aberration correction. Therefore, we could see trends much more abruptly than in other evaluated indicators (Figure 6f). With the exception of point P3, with optimum values at the range between 10 and 25 voxels, there was not a clear interval of preferred nominal thicknesses attending to this indicator, but at least we found again that the worst values were at the extremes of the curves, which is somehow coherent with the rest of the evaluation. No clear preferences were obtained between concave and convex lenses, or between near and distant points.

Energetic overlapping: The values of this indicator are deeply linked to the values of previously commented ones, and therefore the data in Figure 6g summarizes what we have seen. Then, in comparison with the overlaps at the underwater case, we found smaller values and rougher trends. Particularly, we obtained the worst focusing in transcranial at point P3, whereas it was the best focused in water. This great reduction of focusing probably related with the irregularity of the skull region crossed in this case. All the overlaps were between 20% and 45% and the interval of nominal thickness, in which these values were largest, was again between 10 and 25 voxels for both concave and convex lenses at the three points evaluated. Finally, the values obtained for this indicator in transcranial simulations seemed to be notably independent of the transducer-target distance or the type of lens.

### 4.3. Emission in Water at 380 kHz

**Figure 7:**
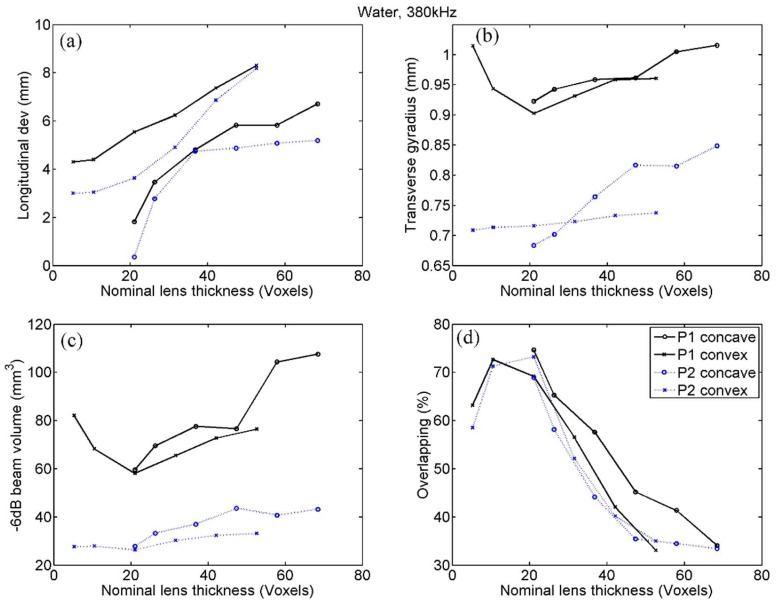
Focusing quality indicators calculated by simulation in water with 380 kHz, at points P1, and P2, for concave (fast) and convex (slow) lenses. Indicators are the following: (**a**) Longitudinal deviation, (**b**) transverse gyradius, (**c**) beam volume and (**d**) energetic overlapping.

Since the results found are very similar to those seen at 760 kHz, the analysis of the graphical information shown in Figure 7 is here faced as a whole. Thus, we can point out that, in the case of concave lenses, all the parameters in both points showed the same type of trend, consisting of a worsening of the indicator quality as the thickness of the lens increased. This is not contradictory with what was seen in the evaluation at 760 kHz, but is due to the fact that the smallest possible lens thickness, conditioned to the value of the water wavelength in the concave lenses, was greater than the values that define the optimum interval. In the case of convex lenses, whose thickness is not limited a priori, two types of trend were shown in the indicators, with one exception. One type of trend, appreciated at beam volume and at both gyradii, was simply flat, so that no conclusion can be drawn from it. For the rest of the indicators, and for both points, the same trend seen for the previous frequency was observed, that is, we found the optimum values of the indicators to be in the range of nominal thickness between 10 and 30 voxels. Although this trend was slight in several indicators, it was clearer for overlapping, which is the most relevant indicator. Finally, it is worth mentioning that (i) in the transverse deviation no significant trend was observed, since its values in the underwater case are only indicative of the numerical error and that (ii) in the longitudinal deviation no clear optimum is appreciated, but we interpret that the indicator improves the smaller the thickness of the lens. This last result could be justified by the fact that the thickness of the lens conditions the position of the transducer to maintain the position of the phase registration curve. Therefore, it seems logical that the change of position of the transducer in the longitudinal axis affects the longitudinal position of the focus, so that this indicator is the only one in which the results indicate that it is preferable to reduce the thickness as much as possible.

### 4.4. Transcranial Emission at 380 kHz

**Figure 8:**
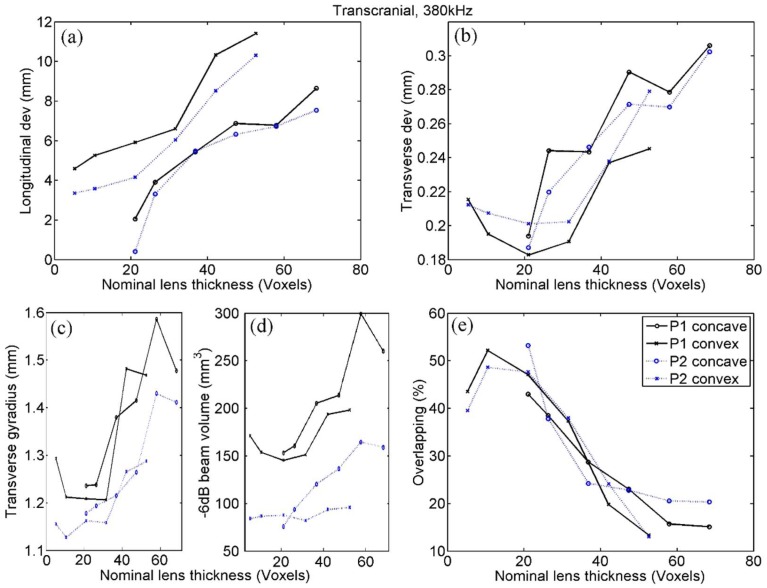
Focusing quality indicators calculated by transcranial simulation with 380 kHz at points P1 and P2, for concave (fast) and convex (slow) lenses. Indicators are as follows: (**a**) Longitudinal and (**b**) transverse deviation, (**c**) transverse gyradius, (**d**) beam volume, and (**e**) energetic overlapping.

The results obtained (Figure 8) are in good agreement with those found in water at the same frequency. Furthermore, the transverse deviation, which is not a significant indicator in water, broadly show the same trend seen in the rest of the parameters. That is, an area of optimum values was between 10 and 30 voxels for convex lenses, while in concave lenses, which do not cover this range, the indicator worsened as the thickness increases. Additionally, noteworthy was the high parallelism of the results found in both transcranial and water cases, which can be justified because the capability of the skull to generate aberrations is less in the case of a longer wavelength.

### 4.5. Experimental Validation

**Figure 9:**
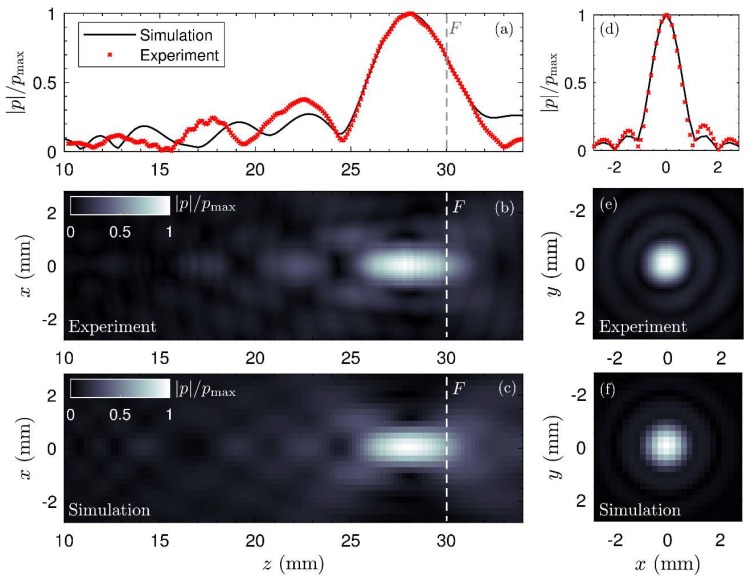
Results of the experimental validation test. (**a**) Experimental (red markers) and simulated (black continuous) normalized pressure field distribution measured at the axis of symmetry. Pressure field distribution in the sagittal plane, *P*(*x*,*z*), obtained (**b**) experimentally and (**c**) numerically. (**d**) Experimental (red markers) and simulated (black continuous) normalized pressure field distribution in the transverse direction *x* measured at the focal spot. Corresponding pressure distributions in the transverse plane, *P*(*x*,*y*), obtained (**e**) experimentally and (**f**) numerically.

The results of the field measurements are summarized in Figure 9. First, the measured field and the corresponding simulation at 1.12 MHz along the axis of symmetry of the lens are shown in Figure 9a. A good agreement was found between the simulation and the experiment. The focal spot has been well described by simulations. Note that the focal spot peaks at *z* = 28.3 mm instead of at *z* = *F* = 30 mm. This is due to the diffraction effects of the wavefront, in accordance with the axial-focal shift expected in any focused source, as described in Reference [48]. The pressure distribution was measured in the sagittal plane, *P*(x,z), and the normalized absolute value of the field is shown in Figure 9b for the experiment and Figure 9c for the simulation. An excellent agreement was found between both fields. We remark that the field showed excellent symmetry as expected by the geometry of the lens. The corresponding transverse field distribution measured at the focal spot is shown in Figure 9d–f for both the simulation and the experiment. The focal spot presented a sharp transverse size smaller than the wavelength (about 1.2 mm). Although small discrepancies were observed at the side-lobes of the pattern, the sharp focus observed experimentally agrees well with simulations, showing that the proposed computational method describes in an accurate manner the expected behavior of the holographic lenses. 

## 5. Discussion 

Although the extension of this study does not allow the quantification of the statistical significance of the results obtained, certain relevant evidence can be extracted, which is discussed below. In order to obtain a conclusive result regarding the precise physical parameters that define the optimum lens, in addition to more similar experiments to increase the statistical significance of the results, it would be necessary to increase the number of types of simulations, with more wavelength values, more distances to the focus point (including points beyond the geometric centre of the transducer), or more lens apertures. Various numerical methods should also be applied, as the wave speed directly affects the stability of the calculation, which limits the reliability of the results for lenses with speeds highly different to the medium, whether faster or slower. 

However, although we cannot determine the exact properties of the optimal lens, the presence of a trend that allows the establishment of an interval of desirable values has been clearly demonstrated by the results obtained. Indeed, as seen in Figure 5, Figure 6, Figure 7 and Figure 8, all the quantitative focusing indicators obtained in the simulations with 760 kHz, for the two types of lens, at any point, and with or without the skull, presented a behaviour regarding the lens thickness that can be expressed as follows. For thicknesses greater than a certain value of about 15 or 20 voxels, the indicators worsen with the increase in thickness, although this relationship is moderate and sometimes even flat (as in the case of the transcranial focal volume in point 2). For thicknesses smaller than 10 or 15 voxels, the dependence between the value of the indicators and the thickness is much more abrupt, so that thicknesses under 10 voxels are clearly discouraged. This trend is manifested in all indicators in the case of slow lenses, since with fast lenses thicknesses smaller than water wavelength cannot be obtained. However, this result could not be conclusive because the bad value of indicators could be partially associated with the numerical error in the propagation inside the lens, given that the number of points per wavelength in extremely thin lenses is less than recommended [32]. In any case, and due to the fact that the impedance value has been fixed to achieve total transparency, which means that there are no internal reflections in the lens, we can assume that the possible numerical error in the phase associated with a path inside the lens as short as 5 or 10 voxels would not have a considerable cumulative effect, but that the origin of the error is precisely the strong discretization of the values of the phase at the exit of the lens associated with the fact that its thickness is defined by such a scarce number of voxels. This hypothesis is reinforced by the fact that in the range of 10 to 15 voxels we can already see, albeit in a moderate way, that lower thickness implies a worse value of the indicators. 

Similar conclusions can be drawn from the analysis of the results obtained with the 380 kHz emission. Fast lenses here are always thicker than the optimum, so it was observed that greater thicknesses directly imply worse indicators; as for slow lenses, we observed the trend commented in the case of 760 Hz with all indicators, with the sole exception of the longitudinal deviation. 

In the simulations at both frequencies certain additional relationships can be seen, which are briefly discussed below. Thus, as point P1 was farther away than P2, higher values of focus size or gyradius were obtained, which is predictable; however, the obtained values of the transverse deviation or the transcranial energetic overlapping were similar at both evaluated points. In water simulations, however, it seems clear that the indicators are better for the farthest point, which makes sense since a farther target implies smoother lens curvatures, which results in (i) the appearance of fewer edges associated with the phase shift 2π and, on the one hand, (ii) a smaller deviation of rays at the boundary of the lens, which is better suited to the thin lens model and additionally activates less energy in form of s-waves. Another foreseeable result is that in the process of passing through the skull, even if the incidence is made with phase correction, energy is dispersed, so that the beam contours are diffuse and indicators obtained are generally worse than that of underwater case.

The simulation with several frequencies, one of which deviated from the usual values in medical treatments, aimed to study whether the optimal thickness values are intrinsic or dependent on the wavelength, given a voxel size. It was observed that with different wavelengths the curves were not displaced in the axis of thicknesses, but that, for both 380 and 760 kHz, the best indicators were in the zone between 10 and 25 voxels thick, regardless of the kind of lens, the distance transducer-target, or the presence or absence of the skull. This result deserves to be highlighted by its relevance in order to generalize the study, as all the magnitudes measured in meters of the problem are related. As an example, if the lens aperture, its radius, the transducer-target distance, and the wavelength were duplicated simultaneously, we would have a mathematically equivalent situation to the initial state (disregarding the effect of absorption), but with a voxel of half the size. Therefore, if we change only some of those lengths, we face different situations. With that said, finding that the ideal range of thickness (in voxel) does not depend on either wavelength, distance, or aperture is a remarkable result. In this same sense, it is worth mentioning that the frequencies used in medical treatments (about 1 MHz) are associated with an optimal lens thickness that can be achieved with polymers of fairly common properties, both in the case of concave and convex lenses, as can be seen in the Table 1 and Table 2. On the other hand, in the case of 380 kHz, not usual in transcranial FUS, it can be seen (Table 2) that no material has the necessary rigidity to reach the optimum thickness of concave lenses since fast lenses have a nominal thickness larger than the wavelength of the sound in the water. This could be solved with slow lenses whose thickness is not limited, although, as is shown below, slow lenses are affected by other inconveniences.

The error associated with the possible difference between the speed of the printed lens and the one assumed in the simulation, represented in the analytical curves of the Figure 3, was not evaluated by the set of simulations. This error presents a notable asymmetry, so that it tends asymptotically to zero on the side of fast lenses, whereas on the side of slow lenses (elastomers) both this error and that related to discretization tend to produce not negligible values. Thus, from the point of view of the focusing quality, we could conclude that lenses “as slow as possible” are discouraged a priori while lenses “as fast as possible” are recommended as long as they have an adequate impedance, as is discussed below.

**Figure 10:**
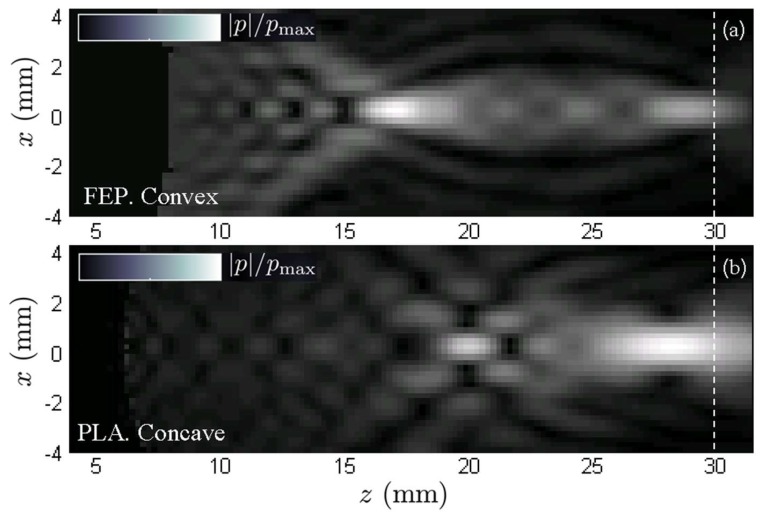
Numerically simulated pressure field distributions in the sagittal plane, *P*(x,z), obtained with two lenses generated from the same particular registered pattern. (**a**) Convex lens made of fluorinated ethylene propylene and (**b**) concave lens made of polylactic acid.

New evidence in favor of rigid lenses can be drawn from the preliminary results of the experimental validation (Figure 10) and its analytical basis is discussed below. Most of the energy is propagated in the form of p-waves, however, s-waves cannot be neglected due to the abrupt curvature often presented by the lenses. Thus, the fact that the s-wave speed is always lower than the p-wave speed affects each type of lens very differently. In slow lenses, the s-wave speed is even slower, which makes the s-waves even more refractive, allowing the generation of secondary foci between the primary and the lens. These foci, by geometric proximity to the source, could eventually concentrate high energy, which is undesirable. In fast lenses, on the other hand, the value of the s-wave speed can be faster than the sound speed at water but also slower. In the first case, the refraction would be lower than in p-waves so that, if a secondary focus were produced, it would be beyond the primary focus accumulating low energy. In the second case (s-wave speed lower than water) the s-wave would present an opposite vergence to the p-wave, so the lens would disperse the energy instead of concentrating it. Both possibilities in fast lenses are clearly favourable with respect to the risks associated with the application of slow lenses. However, if these are made with elastomers with a Poisson’s ratio very close to 0.5, the medium behaviour is practically equivalent to a fluid with a single associated propagation speed, which would somehow validate the use of slow lenses.

The set of simulations aimed to find the range of preferred thicknesses, exclusively accounting for the effect of the polymer wave speeds. However, the important effect of the impedance must be highlighted and is discussed briefly here. From the theoretical point of view, it is simple to establish that its preferred value is equal to that of water, but in practice this cannot always be achieved. In fact, the velocity of the p-waves, *c*_p_, and the acoustic impedance, *z*, are related according to the expression *z* = *ρ_o_c*_p_, where *ρ_o_* is density. Thus, as shown at Table 1, whereas in fast materials the density is practically constant so that an increase of the speed carries implicitly an increase in the impedance, in the slow ones one can find different densities and therefore water normalized acoustic impedances close to the unit. The highest transparencies, nearing 100%, are found with fluorocarbons, such the fluorinated ethylene propylene (FEP). Therefore, from the point of view of energy efficiency, it would be preferable a priori to use slow lenses as long as they are elastomers with unitary water normalized impedances. However, from a strictly focusing-quality point of view, the use of fast lenses is still recommended because, as stated in previous sections about sources of error, by knowing the physical properties of the transducer and backing, the phase error associated with internal reflections in the lens can be completely corrected. In any case, although the phase error associated with impedance is known and can be corrected by designing the lens using the Fabry–Pérot hypothesis [9], the fact that total transparency is only reached when the impedances of lenses and water are equal constitutes a clear handicap for rigid lenses.

An additional aspect to keep in mind is that the transmission from the transducer to the supposedly spherical inner surface of the lens will be notably affected by the fact that 3D printing does not produce a perfect curve, but rather a voxeled Cartesian model. Thus, the contact between the transducer and the lens is not homogeneous, but concentrations of stresses may occur in certain specific vertexes of the lens with the risk of high transmission irregularities. These concentrations will be more pronounced in rigid and crystalline plastics than in deformable and amorphous plastics such as elastomers. Although high impedance gels [49] can soften this effect, we recommend facing its quantitative study in later works. These arguments are no longer valid when emitting by flat transducers, as in the validation experiment.

The choice of a nominal lens thickness associated with a phase shift 2π, not discussed in previous sections, can be justified attending to the results of the simulations. Indeed, they coincide with the thin lens hypothesis assumed, since, disregarding the error associated with the spatial discretization of the lens, the finer the lens the better the focusing. Therefore, even when Fresnel lenses with a 2π phase shift have many areas of abrupt curvature, their use is reasonably justified compared to continuous lenses or lenses with a larger associated phase shift that would be much thicker.

Summarizing, we could say that slow lenses have the advantages of (i) certain independence between p-wave speed and acoustic impedance so that it is possible to simultaneously achieve speeds very different to water with very similar impedances and therefore high transparency and, (ii) in the case of lenses made of elastomers to be used with spherical transducers, an easier mechanical coupling that avoids concentrations of tensions in the micro-vertices of the Cartesian mesh. Fast lenses, on the other hand, have the advantage of (i) a lower risk of energy concentrations in secondary foci associated with s-waves, as well as (ii) a much less vulnerable behaviour related to possible inaccuracies in the manufacturing processes of the lens. Finally, it should be noted that results of the simulations indicate that the range of optimum lens thicknesses given a 3D printer voxel size is coincident for both fast and slow lenses, and notably independent of other factors such as emission frequency, transducer aperture, or transducer-target distance.

Based on the analysis of the results obtained, it may be too ambitious to conclusively establish the best material to produce lenses for FUS treatments, however, as an example, we could highlight certain specific materials. Indeed, although each commercial ultrasound equipment has its own emission frequencies and each treatment may have a different optimum frequency, there is some consensus in applying frequencies around 1 MHz [50]. For this specific frequency, and given the inverse proportionality between the applied frequency and the nominal thickness, we can see that polyetherimide (PEI) and Somos®PerFORM have associated thicknesses of 18 and 11 voxels, respectively, so any material placed between them in Table 1 would be suitable. Therefore, the most recommended polymers would be those with the highest transmission, such as PEI, polyetheretherketone (PEEK), or Standard Grey®. As the frequency of the FUS used in the treatment increases, the required lens thicknesses are lower, so that more materials in the table become adequate. If, for example, a frequency of 1.25 MHz is used, all the materials in the table would have associated thicknesses lower than 21 voxels, as all are acceptable with the exception of extreme values, such as Somos®PerFORM, FEP, or TPU with extremely small associated thicknesses of 8, and 4 voxels respectively. In this case a good choice would be ABS or Vero®Clear with transmissions larger than 0.95. PLA and PSU also show high transmissions and adequate thicknesses for this frequency, but they could generate focusing inaccuracies associated with the printing process (Figure 3) because their p-wave speeds are excessively similar to that of water.

## 6. Conclusions

In this work we looked for the best polymers to 3D print holographic acoustic lenses, particularly for application in the correction of transcranial FUS aberrations. Therefore, although there are many parameters affecting the focusing quality of a lens, some of them affect in a trivial way, so it is simple to establish their desirable values a priori. We can, for example, establish that the desirable material must be homogeneous and with low acoustic absorption. However, there are two main parameters with a non-trivial relationship with quality, as follows: The impedance and the p-wave speed in the lens material. As for the impedance, and attending to focusing quality, any value can be optimal if we know exactly the properties of medium, transducer, and backing. Since their exact values are difficult to know, it is as much as saying that not all impedances values are optimal and the only robust value that does not require the exact knowledge of all agents is an acoustic impedance equal to that of water.

Thus, this study is centered on the effect of the sound speed of the polymer, highly related to the thickness of the lens. In particular, this work proves that there is an optimum range of lens thicknesses, given that analytical models work best for fine lenses, but the discretization in voxels associated with 3D printing is a greater source of error the finer the lens. The set of numerical and experimental evaluations leaves a series of clear evidence. If we divide the materials into three groups of p-wave speeds, such as (i) notably lower than water, (ii) near water, and (iii) notably higher than water, it becomes clear, both from theoretical analyses and simulations, that the second group should be avoided. On the other hand, attending to numerical simulations, optimal lens thicknesses are in the same range, between 10 and 25 voxels, for both slow and fast lenses, although fast lenses have certain advantages.

The main advantages associated with lenses made of polymers with high p-wave speeds are the following: (i) In fast lenses the presence of s-waves contaminates the main focus in a less risky way than in slow lenses, (ii) their behaviour is much more robust against eventual manufacturing errors that result in lenses with a p-wave speed different than expected, and (iii) rigid crystalline polymers employed for fast lenses have smaller acoustic absorptions than more deformable amorphous thermoplastics. Slow lenses, especially if they are made of elastomers in which the formation of s-waves can be neglected, have a higher energy efficiency associated with the value of their impedances, but do not offer clear advantages if the focusing quality is strictly evaluated.

It has been found that the optimum nominal thickness in voxels is significantly independent of frequency. So, if we modify the applied frequency, we must accordingly change the material used to print the lens in order to remain within the optimal range. This is why we cannot conclude with a single preferable polymer, but in the discussion section certain optimal materials for a series of case studies are presented.

Transcranial aberration correction based on 3D printed lenses has been proposed recently as a technology capable of competing with the currently established solution based on phased arrays. The advantages of the 3D lenses are fundamentally related to the lower initial acquisition cost of the equipment, whereas the phased arrays can be more straight forward in their later use. However, apart from practical criteria of ease or cost, scientific criteria regarding the focusing accuracy must be considered when choosing one of these alternatives. In future works we will deal with the exhaustive comparison between the focusing quality achieved by 3D lenses and phased arrays, so the present study was proposed as a necessary step prior to such a comparison between both technologies.

## Figures and Tables

**Table 1:** Acoustical data of a series of 3D printable polymers, assuming for water *c* = 1485 m/s and *z* = 1485 krayl. The value τ represents acoustic transmission on a single layer lens/water. Nominal thickness, *d*, is obtained assuming a frequency of 760 kHz and a voxel size of 0.244 mm. SLA, stereolithography; FDM, fused deposition modelling; FEP, fluorinated ethylene propylene; PEI, polyetherimide; PEEK, polyetheretherketone.

		Dens.	Young	*M*						*d*	*d*
Material	Tech.	(g/cm^3^)	(MPa)	(MPa)		*c* (m/s)	c_rel_	z_rel_	τ	(*λ*)	(voxel)
Somos® PerFORM	SLA	1.61	10500	19830	0.32	3510	2.36	3.81	0.66	1.73	14
Polyamida	FDM	1.36	5000	10710	0.4	2810	1.89	2.57	0.81	2.12	17
Standard Grey®	SLA	1.16	3000	6970	0.41	2440	1.65	1.92	0.9	2.54	20
PEEK	FDM	1.32	3600	7710	0.4	2420	1.63	2.15	0.87	2.59	21
PPS	FDM	1.63	4000	8570	0.4	2290	1.54	2.52	0.81	2.84	23
PEI	FDM	1.27	3200	6380	0.39	2240	1.51	1.92	0.9	2.96	24
VERO® Clear	SLA	1.17	3000	4810	0.35	2030	1.37	1.6	0.95	3.73	30
ABS	FDM	1.09	2500	4420	0.37	2010	1.36	1.48	0.96	3.81	30
PSU	FDM	1.25	2610	4620	0.37	1920	1.29	1.62	0.94	4.4	35
PLA	FDM	1.26	2600	4170	0.35	1820	1.23	1.54	0.95	5.44	44
Silicone	Mold	0.98	150	1320	0.48	1160	0.78	0.77	0.98	3.56	28
FEP	DLP	2.17	500	1900	0.45	930	0.63	1.37	0.98	1.7	14
TPU	FDM	1.1	500	440	0.48	630	0.43	0.47	0.87	0.74	6

**Table 2:** Recommended p-wave speeds and nominal lens thicknesses, *d*, for the experiment and simulation frequencies, assuming that the number of voxels associated with the ideal nominal thickness value, *n_d_*, is 15. The last column shows the minimal value of *n_d_* that can be achieved by the fastest polymer listed in Table 1.

		Experiment	Simulation			Assumed Data
Frequency/kHz		1112	760	380		*c*_water_*=* 1485 m/s
*n_d_*		15	15	15	28	voxel size, dh = 0.244 mm
*d*/mm		3.66	3.66	3.66	6.84	
*d*/*λ*		2.74	1.87	0.94	1.75	Applied equation
Fast lens	*c*_relative_	1.57	2.14	---	2,33	$c_{LENS}=\frac{c_{H_{2}0}}{1\pm \frac{c_{H_{2}0}}{f\cdot n_{d}\cdot dh}}$
	*c*/(m/s)	2340	3180	---	3470	
Slow lens	*c*_relative_	0.73	0.65	0.48	0.64	
	*c*/(m/s)	1090	970	720	940	
